# Next-Generation Imaging Techniques: Functional and Miniaturized Optical Lenses Based on Metamaterials and Metasurfaces

**DOI:** 10.3390/mi12101142

**Published:** 2021-09-23

**Authors:** Dasol Lee, Minkyung Kim, Junsuk Rho

**Affiliations:** 1 Department of Mechanical Engineering, Pohang University of Science and Technology (POSTECH), Pohang 37673, Korea; dasol@yonsei.ac.kr (D.L.); kmk961120@postech.ac.kr (M.K.); 2 Department of Biomedical Engineering, Yonsei University, Wonju 26493, Korea; 3 Department of Chemical Engineering, Pohang University of Science and Technology (POSTECH), Pohang 37673, Korea; 4 POSCO-POSTECH-RIST Convergence Research Center for Flat Optics and Metaphotonics, Pohang 37673, Korea

**Keywords:** metamaterials, metasurfaces, imaging, hyperlens, metalens, miniaturized imaging devices

## Abstract

A variety of applications using miniaturized optical lenses can be found among rapidly evolving technologies. From smartphones and cameras in our daily life to augmented and virtual reality glasses for the recent trends of the untact era, miniaturization of optical lenses permits the development of many types of compact devices. Here, we highlight the importance of ultrasmall and ultrathin lens technologies based on metamaterials and metasurfaces. Focusing on hyperlenses and metalenses that can replace or be combined with the existing conventional lenses, we review the state-of-art of research trends and discuss their limitations. We also cover applications that use miniaturized imaging devices. The miniaturized imaging devices are expected to be an essential foundation for next-generation imaging techniques.

## 1. Introduction and Need for Functional and Miniaturized Imaging Devices

Miniaturization of optical lenses is emerging as an essential task as technology evolves. The development of compact devices such as mobile phones, cameras, or augmented/virtual reality devices demands miniaturization of lenses down to sub-micrometer scales. In this regime, a new type of lens other than conventional convex or concave lenses is required for two reasons. First, the fabrication of conventional curved lenses using the traditional cutting or curving process is challenging. Second, geometrical optics that underpins the light focusing on those conventional lenses fails to work in this regime as the wavelength of interest is not sufficiently larger than the lens dimension and electromagnetic wave optics should be considered instead.

For these reasons, attention has focused on use of metamaterials and metasurfaces that can implement various functions and can be miniaturized. Metamaterials are artificially engineered optical materials that are designed to exhibit unconventional properties. Metasurfaces, on the other hand, are two-dimensional counterparts of metamaterials and can be used to avoid three-dimensional nanofabrication and to minimize the optical losses by shortening the optical path length. Propagation of light is determined by the geometrical structure, dimensions and arrangement of the subwavelength-scale constituents of the metamaterials. Thus, appropriately-designed metamaterials offer unprecedented abilities to control the properties of light, including trajectory, wavefront, polarization and phase [1,2,3,4,5,6,7,8,9,10,11,12,13,14,15,16,17]. Naturally, metamaterials and metasurfaces have been evaluated as replacements for conventional lenses to focus light [18,19]. Here, we focus on two types of metamaterials-based lenses. First, a hyperlens is a metamaterial-based lens [20,21,22,23,24,25]. It consists of multilayered metal and dielectrics in cylindrical or spherical geometry. A hyperlens has hyperbolic shape of dispersion, which enables access to subdiffraction features that have arbitrarily high spatial frequencies and transfer of evanescent waves that contain super-resolution features of an object to a far-field. In addition, the resolution below the diffraction limit, hyperlenses can be applied to a miniaturized imaging device by using the latest nanofabrication techniques. Dimensions of the hyperlenses are typically a few micrometers, so they can be easily implemented in conventional optics compactly or used as ultrasmall lensing devices.

Secondly, a metalens is a metasurface-based lens [18,19,26,27,28]. The metalens consists of two-dimensional nanopattern that has deep subwavelength thickness and is fabricated using metals or high-refractive-index dielectric materials. The main focusing mechanism of the metalenses is a spatially gradient phase gain that is determined by the geometry of nanostructures. A metalens that supports a gradient phase profile that is equal to the phase accumulation of the conventional curved lenses can effectively focus light despite ultrasmall thickness. Furthermore, metalenses can be combined with conventional optical systems, or multiple metalenses can be stacked for better performance without making the whole system bulky.

Increased portability and easy implementation to conventional optics as a consequence of the ultrasmall size are distinct advantages of hyperlenses and metalenses that distinguish them from the conventional optical technologies. These metamaterials-based lenses will allow further miniaturization of research equipment including microscopes as well as practical optical devices.

## 2. State-of-the-Art of Metamaterial-Based Imaging Techniques

### 2.1. Hyperlens

The high spatial-frequency components supported by the hyperbolic dispersion provide a direct route toward super-resolution imaging. Additionally, the curved geometry compresses the wave vector of light inside the hyperlens according to the angular momentum conservation law, resulting in a conversion of the evanescent waves to propagating waves as well as the magnification of the images. The concept of the hyperlens was first proposed theoretically in 2006 [22,23,29]. The light propagation inside the hyperlens has been studied using classical optics, an effective medium theory [22,29] and using semiclassical description [30]. In principle, spatial resolution of an ideal hyperlens is arbitrarily large, as implied by the open isofrequency contour, but the finite thickness of the multilayer and fabrication imperfection in evaporating thin film cause violations of effective medium theory at a high wave vector and limit the experimentally available resolution.

**Figure 1:**
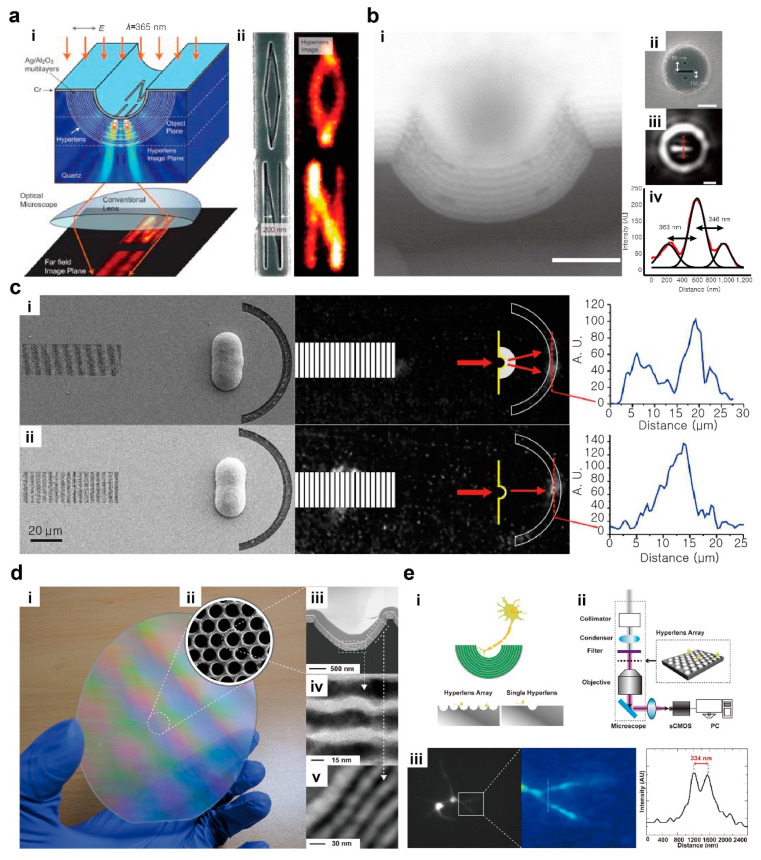
Hyperlens. (**a**) One-dimensional super-resolution imaging that uses a cylindrical hyperlens. (**i**) Schematic of cylindrical hyperlens and (**ii**) simulation result of subdiffraction-limited object. Reprinted with permission from ref. [21], AAAS. (**b**) Two-dimensional super-resolution imaging using a spherical hyperlens. (**i**) A scanning electron microscope (SEM) image of the spherical hyperlens. Measured images of object having two 100 nm size dots and a 100 nm bar through (**ii**) SEM and (**iii**) hyperlens. All scale bars shown in (**i**)–(**iii**) are 500 nm. (**iv**) Cross-sectional analysis result of image by captured hyperlens. Reprinted with permission from ref. [24], Springer Nature. (**c**) Non-resonant, broadband hyperlens. Experimental results of sample (**i**) with hyperlens and (**ii**) without hyperlens. Reprinted with permission from ref. [31], Springer Nature. (**d**) A large-scale hyperlens fabricated by nanoimprinting. (**i**) Photograph, (**ii**) SEM and (**iii**)–(v) TEM images of hyperlens with different magnification. (**e**) A biomolecular imaging using a hyperlens array. (**i**) Concept of positioning samples on a hyperlens array and (**ii**) hyperlens implemented imaging setup. (**iii**) Captured neuron image. Reprinted with permission from ref. [32], ACS.

The theoretical prediction has led to the explosive investigation towards the experimental demonstration of hyperlens-assisted super-resolution imaging [20,21,24]. Early hyperlenses had a cylindrical shape (Figure 1a) that allows super-resolution imaging along one spatial dimension. Silver and aluminum oxide have been used for hyperlenses operating in the ultraviolet regime [20,21]. Subdiffraction-limited resolution of 130 nm [21] and 125 nm [20] have been achieved at wavelength *λ* = 365 nm. Later, two-dimensional super-resolution imaging at visible wavelength was demonstrated by using a spherical hyperlens (Figure 1b). The spherical geometry dictates the conservation of angular momentum along two spatial dimensions and accordingly enables super-resolution imaging along two dimensions. A hyperlens composed of silver and titanium oxide deposited alternatingly on a hemisphere geometry resolved two-dimensional subdiffraction features of 160 nm separation at *λ* = 410 nm.

Although these hyperlenses have the clear advantages of far-field super-resolution capability and compatibility with conventional optics, the multilayered geometry limits the operating wavelength of the super-resolution imaging near the metal plasma frequency as a result of resonant effective permittivity. To overcome this limitation, a radial fan-shaped hyperlens has been proposed as a non-resonant alternative (Figure 1c) [31]. It supports broad bandwidth ranging at 500 ≤ *λ* ≤ 1000 nm theoretically and has achieved low loss super-resolution imaging at *λ* = 780 nm. In addition, non-resonant design, the high optical losses of the hyperlens can be alleviated by adopting new materials such as natural hyperbolic materials [33], semiconductors and transparent conducting oxides [34].

The curved geometry of the hyperlens sometimes imposes restrictions in practical applications. Thus, to improve the practicality and compatibility of the hyperlens, a planar hyperlens has been proposed theoretically, by using transformation optics [35,36,37]. While the planar hyperlens is favorable in implementation to conventional optics, use of planar slabs to mimic the light propagation in a curved geometry complicates the inner geometry. For example, planar hyperlenses entail the curved interfaces between the metal and dielectrics [35,37] or a spatially-varying thickness [36], these shapes require complicated fabrication techniques and have not been experimentally realized so far.

As an alternative, a scalable fabrication technique for large-area hyperlens arrays has been proposed [38]. The previous hyperlens had a single pattern of a few micrometer dimension, which requires a precise control of sample placement. This sensitive sample-positioning step hinders practical implementation of the hyperlens. In contrast, a wafer-scale array of densely packed hyperlenses in a hexagonal pattern fabricated using nanoimprint lithography eliminates such a burden (Figure 1d). As a consequence, a subdiffraction imaging of a biomolecular sample has been achieved with a resolution of 151 nm at *λ* = 410 nm (Figure 1e) [32].

### 2.2. Metalens

**Figure 2:**
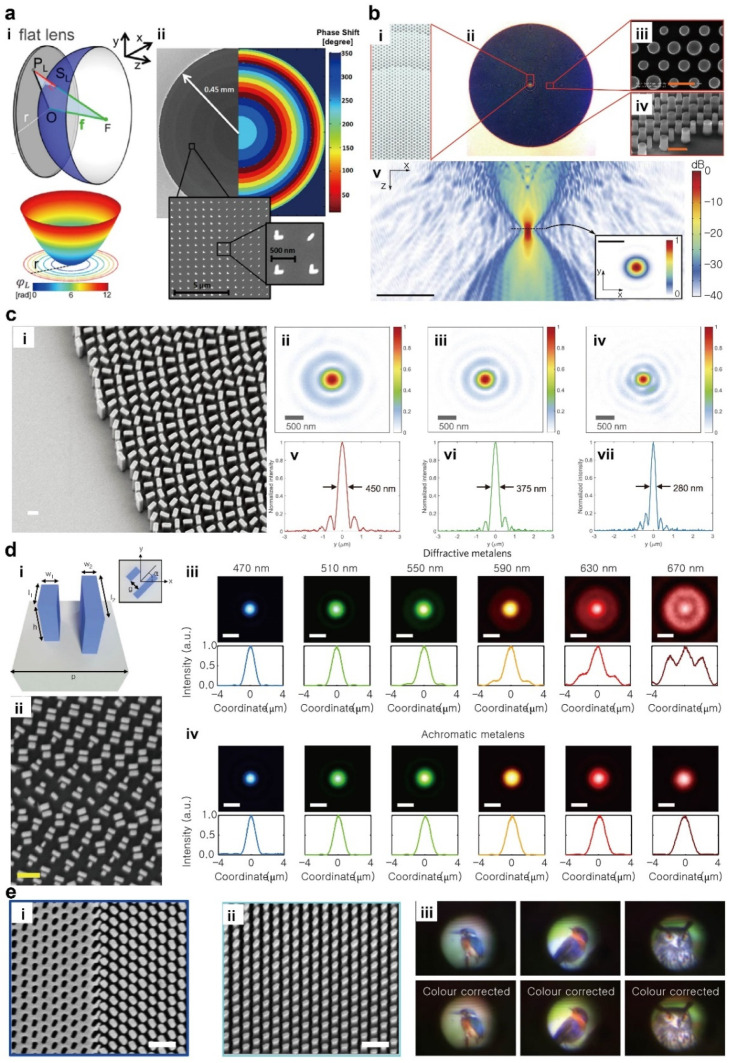
Metalens. (**a**) A hyperbolic phase profile of a metalens. (**i**) Hyperbolic phase profile to focus a plane wave to a focal spot. (**ii**) Discretized phase profile for realistic sample. Reprinted with permission from ref. [47], ACS. (**b**). A resonant dielectric metalens. (**i**) Schematic (**ii**) optical microscope image and (**iii**,**iv**) SEM images of a fabricated metalens. Scale bars are 1 μm. (**v**) Electric energy density. Scale bars are 20 μm in the main figure and 2 μm in the inset. Reprinted with permission from ref. [49], Springer Nature. (**c**) A non-resonant metalens based on Pancharatnam-Berry phase. (**i**) SEM image of the fabricated metalens. Scale bar is 300 nm. Measured focal spot intensity profile at different wavelength: (**ii**) 660 nm, (**iii**) 532 nm and (**iv**) 405 nm. (**v**)–(**vii**) Corresponding cross-sectioned intensity of focal spots. Reprinted with permission from ref. [46], AAAS. (**d**) A broadband achromatic metalens. (**i**) Schematic and (**ii**) SEM image of a metalens element. Scale bar is 500 nm. (**iii**,**iv**) Intensity profiles of diffractive and achromatic metalenses, respectively. Reprinted with permission from ref. [56], Springer Nature. (**e**) Experimental verification of achromatic metalenses. SEM images of (**i**) Babinet structures and (**ii**) nanopillars region from the fabricated achromatic metalens. Scale bars are 500 nm. (**iii**) Captured images using fabricated achromatic metalens. Reprinted with permission from ref. [57], Springer Nature.

A metalens is a flat lens that uses a metasurface to replace the bulk, curved dielectric lenses in nanoscale [18,19]. The operating mechanism of the metalens has a long history that goes back to diffractive optics [39,40,41,42,43,44,45]. Instead of a curved geometry that yields spatially-varying phase by controlling the optical path length, the metalens modulates the phase profile in the whole 2π phase space and reshapes the wavefront by using nanoscale scatterers [2,46]. A metalens that has a hyperbolic phase profile (Figure 2a) [47,48],

(1) $$\phi \left(r\right)=-\frac{2\pi }{\lambda }\left(\sqrt{r^{2}+f^{2}}-f\right),\;$$

where $r=\sqrt{x^{2}+y^{2}}$ is a radial position and *f* is a focal length, focuses normally-incident light with a planar wavefront as a conventional refractive lens with a focal length of *f* does. More specifically, the phase can be controlled resonantly by using plasmonic, Mie or Fabry–Ferot resonance [49,50,51], or non-resonantly by using geometric phases [52,53,54].

The resonant metalens uses a geometrical shape and dimensions, materials and arrangement of the subwavelength scatterers that are engineered to achieve the desired phase profile. A properly designed metalens focuses monochromatic light to a subwavelength focal spot (Figure 2b). The resonant nature inherently limits the bandwidth to a narrow range but requires relatively small aspect ratio, which makes the resonant metalenses more productive in comparison to the non-resonant ones.

On the other hand, the non-resonant metalenses rely on nanopatterns with high aspect ratio to effectively modulate the phase gain without accompanying resonance. Nevertheless, broad bandwidth and high efficiency have made the non-resonant metalenses appealing candidates for practical lensing applications. The phase modulation of the non-resonant metalenses can also be achieved by tuning the geometries of the nanoscatters, or more simply by rotating anisotropic scatterers [55]. For the latter case, spatially-inhomogeneous orientations of the scatterers assign the desired spatial profile of a geometric phase accumulated in a Poincare sphere, the so-called Pancharatnam–Berry phase (Figure 2c).

To improve the practicality, efforts toward efficient metalenses have been made naturally. Plasmonic metalenses generally suffer from high absorption arising from the intrinsic metallic properties [58]. Thus, one solution to increase the efficiency is to use dielectric materials that have high refractive index and low absorption coefficient simultaneously at a target wavelength [59,60,61,62]. Furthermore, material choice of the metalenses is a critical factor that determines their operating wavelength and efficiency. Thus, metalenses for different target wavelengths generally consist of different materials. Representative dielectric materials include hafnium oxide and aluminum nitride for the ultraviolet [63,64], titanium dioxide and gallium nitride for the visible [65,66] and germanium and silicon for the infrared [67,68] regimes.

A metalens that is designed to have the hyperbolic phase profile (Equation (1)) entails several monochromatic aberrations. However, correction of monochromatic aberration is essential for high numerical aperture, which in turn increases the focusing efficiency and widens the field of view. The off-axis aberration that appears under an obliquely incident light can be eliminated by superposing sinusoidal corrections on the hyperbolic phase profile [69]. Use of a metalens on an aplanatic substrate has been studied theoretically to remove coma aberration and spherical aberration [70]. A double metalens, one side of which is an aperture lens and the other side is a focusing lens, has been proposed to alleviate spherical aberration [71]. Metalenses with monochromatic aberration correction have been designed using optimization methods such as topology optimization [72,73,74].

Chromatic aberration correction is another important procedure to develop a metalens that operates over a wavelength regime. The focusing effect of a metalens that is designed for a single target wavelength generally degrades as it deviates from the target wavelength. Thus, correction terms should use a Taylor series expansion such as

(2) $$\phi \left(r,\omega \right)=\phi \left(r,\omega_{t}\right)+{\frac{\partial \phi }{\partial \omega }|}_{\omega =\omega_{t}}\left(\omega -\omega_{t}\right)+{\frac{1}{2}\frac{\partial^{2}\phi }{\partial \omega^{2}}|}_{\omega =\omega_{t}}{\left(\omega -\omega_{t}\right)}^{2}+O\left(\omega^{3}\right),\;$$

where $\omega_{t}$ is a target angular frequency. The first term on the right-hand side corresponds to the spherical wavefront as Equation (1) states. The derivatives in the second and third terms are a group delay and group delay dispersion respectively, which lead directly to the chromatic aberration [72]. Equation (2) implies that an ideal, aberration-free metalens should satisfy a series of conditions of phase, group delay and group delay dispersion. Metalenses provide a large structural degree-of-freedom in that geometry, dimension, arrangement and materials of the nanoscatterers as well as those of the substructures that consist of a unit cell affect the phase and its derivative.

An achromatic metalens that operates at 470 ≤ *λ* ≤ 670 nm has been demonstrated by independently controlling the phase, group delay and group delay dispersion by using the design principle in Equation (2) (Figure 2d) [56]. Similarly, a broadband achromatic metalens operating over a half of the visible regime has been proposed by using a judiciously designed metalens (Figure 2e) [57]. The efforts towards the metalens beyond the monochromatic operation have been also made for several discrete wavelengths in the visible [72,73] or in the near-infrared [52]. The broad bandwidth has been also achieved by using plasmonic materials despite their strong material dispersion, by compensating for it by using structure dispersion of surface plasmon polaritons [74]. To tackle the series of phase derivatives, several numerical optimization and inverse design methods have been applied to design achromatic metalenses [75,76,77].

Despite its advantages, a metalens that exhibits only fixed functionalities after fabrication is not desirable for applications. Thus, intensive attempts have been made to develop an active metalens, whose optical responses can be reconfigured for target applications at will. A tuning of focal lengths of metalenses has been demonstrated by applying mechanical strain [78,79,80] or displacement [81] or by using phase transition materials [82,83]. Metasurfaces whose two-dimensional optical properties can be assigned and removed using femtosecond laser in a non-volatile and reversible manner provide a new path towards multifocal lensing techniques [84,85].

## 3. Challenges and Perspectives

Metamaterials and metasurfaces provide a new paradigm to replace optical components and systems. However, practical uses of these technologies require development of methods to greatly reduce the production cost of optical components and to manufacture miniaturized components by utilizing equipment that is already used in the semiconductor industry. Several alternatives to fabricate metamaterials and metasurfaces at low cost are emerging. Nanoimprint lithography and deep UV lithography are being considered because they have the advantages of high speed, low cost and compatibility with mass production [78,86,87,88,89].

Detailed challenges and limitations for the two lithography techniques also need to be addressed. For hyperlenses to be practically usable in imaging, their efficiency and operating wavelength range should be increased. A simple and inexpensive method to fabricate large-scale hyperlens would also be an advantage [32]. So far, research on metalenses has focused on ways to overcome the following limitations. First, metalenses are less efficient than traditional lenses. Metalenses do not transmit as much light as the traditional lens, but to be used as a lens of an imaging system that can acquire a clear image, they must be able to utilize most of the incident light. Second, metalenses have a small diameter to capture a sufficient amount of light. This means that to acquire high-quality images, the lens should be large. Third, many optical systems that use metalenses also use additional optical components to process unmodulated signals; this approach makes the entire system complex and bulky and reduces efficiency. Therefore, development of ideal metalenses requires optimization of the diameter and design of the lens to achieve high efficiency and high numerical aperture. In addition to being more practical, metalenses should have polarization-independent characteristics. Furthermore, aberration-free optical systems operating in broad wavelength range should be developed.

Nevertheless, miniaturized lenses and applications will lead to size reduction and simplification of devices in a few years. Research that can increase the efficiency of lenses by exploration of various materials [90] and fabrication conditions is being actively conducted [91]. Establishment of silicon-deposition conditions to minimize optical loss and development of methods to manufacture lenses with larger diameters than now are expected to expedite commercialization of the technology. Potential applications as imaging sensors and systems and diagnostic tools are of great interest to many companies and governments.

Thus, recently reported results are promising in that they have reduced the complexity of sophisticated optical systems and have opened up a wide range of possible applications. Development of hyperlenses has shown the possibility of producing low-cost, large-area devices by large-scale fabrication [32], but research is pursuing innovation by use of various materials to widen the operating wavelength range and increase the efficiency; examples include investigation of organic hyperbolic metamaterials that have low loss or photostability [92]. The use of ultra-small, ultra-high-resolution functional materials has the potential to develop portable devices that can measure various samples with increased efficiency and that can diagnose diseases and identify viruses.

Metalenses also show strong potential for combination with other industrial fields. For example, the lenses are actively being applied to compact and high-resolution microscopes [93,94,95] and to ultra-small optical devices that can be used in virtual and augmented reality [96,97]. Although active metalenses whose functionalities can be manipulated under external stimuli have been demonstrated, considerable efforts still remain for the development of genuine active metalenses for real-world applications.

In addition, they are also showing the possibility of use in functional optical devices that are complicated to implement with traditional optical lenses; examples include a full-Stokes polarization camera [98], a depth-sensing camera that uses multi-focal length metalenses [99,100] and varifocal metalenses [83,101,102,103,104]. Other technologies of organic light-emitting diodes [105], wearable optical devices [106] are also being combined with original industries.

In this opinion, we have focused on the potential of miniaturized lenses based on metamaterials or metasurfaces, with a special focus on hyperlenses and metalenses. These are expected to open new avenues in the construction of new optical devices. Use of metamaterials or metasurfaces may enable encoding of various functions and implementation of miniaturized optical devices. This capability will lead to improvements in optics and systems and will enable the development of single devices that can significantly reduce the volume and cost of existing imaging devices and electronic devices.

## References

[1] Tsilipakos O., Koschny T., Soukoulis C.M. (2018). Antimatched electromagnetic metasurfaces for broadband arbitrary phase manipulation in reflection. ACS Photonics.

[2] Yu N., Genevet P., Kats M.A., Aieta F., Tetienne J.-P., Capasso F., Gaburro Z. (2011). Light propagation with phase discontinuities: Generalized laws of reflection and refraction. Science.

[3] Kruk S., Hopkins B., Kravchenko I.I., Miroshnichenko A., Neshev D.N., Kivshar Y.S. (2016). Invited Article: Broadband highly efficient dielectric metadevices for polarization control. APL Photonics.

[4] Pfeiffer C., Grbic A. (2013). Metamaterial Huygens’ surfaces: Tailoring wave fronts with reflectionless sheets. Phys. Rev. Lett..

[5] Tsilipakos O., Tasolamprou A.C., Koschny T., Kafesaki M., Economou E.N., Soukoulis C.M. (2018). Pairing toroidal and magnetic dipole resonances in elliptic dielectric rod metasurfaces for reconfigurable wavefront manipulation in reflection. Adv. Opt. Mater..

[6] Huang C., Zhang C., Yang J., Sun B., Zhao B., Luo X. (2017). Reconfigurable metasurface for multifunctional control of electromagnetic waves. Adv. Opt. Mater..

[7] Dastmalchi B., Tassin P., Koschny T., Soukoulis C.M. (2014). Strong group-velocity dispersion compensation with phase-engineered sheet metamaterials. Phys. Rev. B.

[8] Khaliq H.S., Kim I., Kim J., Oh D.K., Zubair M., Riaz K., Mehmood M.Q., Rho J. (2021). Manifesting Simultaneous Optical Spin Conservation and Spin Isolation in Diatomic Metasurfaces. Adv. Opt. Mater..

[9] Kim I., Jang J., Kim G., Lee J., Badloe T., Mun J., Rho J. (2021). Pixelated bifunctional metasurface-driven dynamic vectorial holographic color prints for photonic security platform. Nat. Commun..

[10] Kim J., Yang Y., Badloe T., Kim I., Yoon G., Rho J. (2021). Geometric and physical configurations of meta-atoms for advanced metasurface holography. InfoMat.

[11] Kim I., Jeong H., Kim J., Yang Y., Lee D., Badloe T., Kim G., Rho J. (2021). Dual-Band Operating Metaholograms with Heterogeneous Meta-Atoms in the Visible and Near-Infrared. Adv. Opt. Mater..

[12] Jung C., Kim G., Jeong M., Jang J., Dong Z., Badloe T., Yang J.K.W., Rho J. (2021). Metasurface-Driven Optically Variable Devices. Chem. Rev..

[13] Naveed M.A., Ansari M.A., Kim I., Badloe T., Kim J., Oh D.K., Riaz K., Tauqeer T., Younis U., Saleem M. (2021). Optical spin-symmetry breaking for high-efficiency directional helicity-multiplexed metaholograms. Microsyst. Nanoeng..

[14] Kim I., Martins R.J., Jang J., Badloe T., Khadir S., Jung H.-Y., Kim H., Kim J., Genevet P., Rho J. (2021). Nanophotonics for light detection and ranging technology. Nat. Nanotechnol..

[15] Yoon G., Rho J. (2021). MAXIM: Metasurfaces-oriented electromagnetic wave simulation software with intuitive graphical user interfaces. Comput. Phys. Commun..

[16] Badloe T., Lee J., Seong J., Rho J. (2021). Tunable Metasurfaces: The Path to Fully Active Nanophotonics. Adv. Photonics Res..

[17] Badloe T., Kim I., Kim Y., Kim J., Rho J. (2021). Electrically Tunable Bifocal Metalens with Diffraction-Limited Focusing and Imaging at Visible Wavelengths. Adv. Sci..

[18] Lalanne P., Chavel P. (2017). Metalenses at visible wavelengths: Past, present, perspectives. Laser Photonics Rev..

[19] Khorasaninejad M., Capasso F. (2017). Metalenses: Versatile multifunctional photonic components. Science.

[20] Lee H., Liu Z., Xiong Y., Sun C., Zhang X. (2007). Development of optical hyperlens for imaging below the diffraction limit. Opt. Express.

[21] Liu Z., Lee H., Xiong Y., Sun C., Zhang X. (2007). Far-field optical hyperlens magnifying sub-diffraction-limited objects. Science.

[22] Salandrino A., Engheta N. (2006). Far-field subdiffraction optical microscopy using metamaterial crystals: Theory and simulations. Phys. Rev. B.

[23] Jacob Z., Alekseyev L.V., Narimanov E. (2006). Optical hyperlens: Far-field imaging beyond the diffraction limit. Opt. Express.

[24] Rho J., Ye Z., Xiong Y., Yin X., Liu Z., Choi H., Bartal G., Zhang X. (2010). Spherical hyperlens for two-dimensional sub-diffractional imaging at visible frequencies. Nat. Commun..

[25] Lee D., Kim M., So S., Kim I., Yoon G., Kim K., Rho J. (2017). Demonstration of a Hyperlens-integrated Microscope and Super-resolution Imaging. J. Vis. Exp..

[26] Moon S.-W., Kim Y., Yoon G., Rho J. (2020). Recent progress on ultrathin metalenses for flat optics. iScience.

[27] Rho J. (2020). Metasurfaces: Subwavelength nanostructure arrays for ultrathin flat optics and photonics. MRS Bull..

[28] Lee D., Gwak J., Badloe T., Palomba S., Rho J. (2020). Metasurfaces-based imaging and applications: From miniaturized optical components to functional imaging platforms. Nanoscale Adv..

[29] Wood B., Pendry J.B., Tsai D.P. (2006). Directed subwavelength imaging using a layered metal-dielectric system. Phys. Rev. B.

[30] Jacob Z., Alekseyev L.V., Narimanov E. (2007). Semiclassical theory of the hyperlens. J. Opt. Soc. Am. A.

[31] Sun J., Shalaev M.I., Litchinitser N.M. (2015). Experimental demonstration of a non-resonant hyperlens in the visible spectral range. Nat. Commun..

[32] Lee D., Kim Y.D., Kim M., So S., Choi H.-J., Mun J., Nguyen D.M., Badloe T., Ok J.G., Kim K. (2018). Realization of Wafer-Scale Hyperlens Device for Sub-diffractional Biomolecular Imaging. ACS Photonics.

[33] Li P., Lewin M., Kretinin A.V., Caldwell J.D., Novoselov K.S., Taniguchi T., Watanabe K., Gaussmann F., Taubner T. (2015). Hyperbolic phonon-polaritons in boron nitride for near-field optical imaging and focusing. Nat. Commun..

[34] Naik G.V., Shalaev V.M., Boltasseva A. (2013). Alternative plasmonic materials: Beyond gold and silver. Adv. Mater..

[35] Han S., Xiong Y., Genov D., Liu Z., Bartal G., Zhang X. (2008). Ray optics at a deep-subwavelength scale: A transformation optics approach. Nano Lett..

[36] So S., Rho J. (2019). Geometrically flat hyperlens designed by transformation optics. J. Phys. D Appl. Phys..

[37] Cheng B.H., Ho Y.Z., Lan Y.-C., Tsai D.P. (2012). Optical hybrid-superlens hyperlens for superresolution imaging. IEEE J. Sel. Top. Quantum Electron..

[38] Byun M., Lee D., Kim M., Kim Y., Kim K., Ok J.G., Rho J., Lee H. (2017). Demonstration of nanoimprinted hyperlens array for high-throughput sub-diffraction imaging. Sci. Rep..

[39] Kock W.E. (1948). Metallic delay lenses. Bell Syst. Tech. J..

[40] d’Auria L., Huignard J.P., Roy A.M., Spitz E. (1972). Photolithographic fabrication of thin film lenses. Opt. Commun..

[41] Chen F.T., Craighead H.G. (1996). Diffractive lens fabricated with mostly zeroth-order gratings. Opt. Lett..

[42] Lalanne P., Astilean S., Chavel P., Cambril E., Launois H. (1999). Design and fabrication of blazed binary diffractive elements with sampling periods smaller than the structural cutoff. J. Opt. Soc. Am. A.

[43] Lee M.-S.L., Lalanne P., Rodier J.-C., Chavel P., Cambril E., Chen Y. (2002). Imaging with blazed-binary diffractive elements. J. Opt. A Pure Appl. Opt..

[44] Jiang W.X., Qiu C., Han T.C., Cheng Q., Ma H.F., Zhang S., Cui T.J. (2013). Broadband All-Dielectric Magnifying Lens for Far-Field High-Resolution Imaging. Adv. Mater..

[45] Jiang W.X., Ge S., Han T., Zhang S., Mehmood M.Q., Qiu C., Cui T.J. (2016). Shaping 3D Path of Electromagnetic Waves Using Gradient-Refractive-Index Metamaterials. Adv. Sci..

[46] Khorasaninejad M., Chen W.T., Devlin R.C., Oh J., Zhu A.Y., Capasso F. (2016). Metalenses at visible wavelengths: Diffraction-limited focusing and subwavelength resolution imaging. Science.

[47] Aieta F., Genevet P., Kats M.A., Yu N., Blanchard R., Gaburro Z., Capasso F. (2012). Aberration-free ultrathin flat lenses and axicons at telecom wavelengths based on plasmonic metasurfaces. Nano Lett..

[48] Chen X., Huang L., Mühlenbernd H., Li G., Bai B., Tan Q., Jin G., Qiu C.-W., Zhang S., Zentgraf T. (2012). Dual-polarity plasmonic metalens for visible light. Nat. Commun..

[49] Arbabi A., Horie Y., Ball A.J., Bagheri M., Faraon A. (2015). Subwavelength-thick lenses with high numerical apertures and large efficiency based on high-contrast transmitarrays. Nat. Commun..

[50] Anzan-Uz-Zaman M., Song K., Lee D.-G., Hur S. (2020). A novel approach to Fabry–Pérot-resonance-based lens and demonstrating deep-subwavelength imaging. Sci. Rep..

[51] Wang S., Wu P.C., Su V.-C., Lai Y.-C., Chu C.H., Chen J.-W., Lu S.-H., Chen J., Xu B., Kuan C.-H. (2017). Broadband achromatic optical metasurface devices. Nat. Commun..

[52] Khorasaninejad M., Chen W.-T., Oh J., Capasso F. (2016). Super-dispersive off-axis meta-lenses for compact high resolution spectroscopy. Nano Lett..

[53] Yang H., Li G., Cao G., Zhao Z., Yu F., Chen X., Lu W. (2017). Polarization-independent metalens constructed of antennas without rotational invariance. Opt. Lett..

[54] Piccirillo B., Picardi M.F., Marrucci L., Santamato E. (2017). Flat polarization-controlled cylindrical lens based on the Pancharatnam–Berry geometric phase. Eur. J. Phys..

[55] Nikolova L., Ramanujam P.S. (2009). Polarization Holography.

[56] Chen W.T., Zhu A.Y., Sanjeev V., Khorasaninejad M., Shi Z., Lee E., Capasso F. (2018). A broadband achromatic metalens for focusing and imaging in the visible. Nat. Nanotechnol..

[57] Wang S., Wu P.C., Su V.-C., Lai Y.-C., Chen M.-K., Kuo H.Y., Chen B.H., Chen Y.H., Huang T.-T., Wang J.-H. (2018). A broadband achromatic metalens in the visible. Nat. Nanotechnol..

[58] Ni X., Ishii S., Kildishev A.V., Shalaev V.M. (2013). Ultra-thin, planar, Babinet-inverted plasmonic metalenses. Light Sci. Appl..

[59] Chen J., Zhang F., Li Q., Wu J., Wu L. (2018). A high-efficiency dual-wavelength achromatic metalens based on Pancharatnam-Berry phase manipulation. Opt. Express.

[60] Genevet P., Capasso F., Aieta F., Khorasaninejad M., Devlin R. (2017). Recent advances in planar optics: From plasmonic to dielectric metasurfaces. Optica.

[61] West P.R., Stewart J.L., Kildishev A.V., Shalaev V.M., Shkunov V.V., Strohkendl F., Zakharenkov Y.A., Dodds R.K., Byren R. (2014). All-dielectric subwavelength metasurface focusing lens. Opt. Express.

[62] Khorasaninejad M., Zhu A.Y., Roques-Carmes C., Chen W.T., Oh J., Mishra I., Devlin R.C., Capasso F. (2016). Polarization-insensitive metalenses at visible wavelengths. Nano Lett..

[63] Guo L., Hu Z., Wan R., Long L., Li T., Yan J., Lin Y., Zhang L., Zhu W., Wang L. (2019). Design of aluminum nitride metalens for broadband ultraviolet incidence routing. Nanophotonics.

[64] Zhang C., Divitt S., Fan Q., Zhu W., Agrawal A., Lu Y., Xu T., Lezec H.J. (2020). Low-loss metasurface optics down to the deep ultraviolet region. Light Sci. Appl..

[65] Khorasaninejad M., Shi Z., Zhu A.Y., Chen W.-T., Sanjeev V., Zaidi A., Capasso F. (2017). Achromatic metalens over 60 nm bandwidth in the visible and metalens with reverse chromatic dispersion. Nano Lett..

[66] Huang B., Bai W., Jia H., Han J., Guo P., Wu J., Yang J. (2020). Multifocal co-plane metalens based on computer-generated holography for multiple visible wavelengths. Results Phys..

[67] Wang A., Chen Z., Dan Y. (2019). Planar metalenses in the mid-infrared. AIP Adv..

[68] Wang S., Sun X., Chen D., Wang S., Qi Y., Wu F. (2020). The investigation of height-dependent meta-lens and focusing properties. Opt. Commun..

[69] Kalvach A., Szabó Z. (2016). Aberration-free flat lens design for a wide range of incident angles. J. Opt. Soc. Am. B.

[70] Aieta F., Genevet P., Kats M., Capasso F. (2013). Aberrations of flat lenses and aplanatic metasurfaces. Opt. Express.

[71] Groever B., Chen W.T., Capasso F. (2017). Meta-lens doublet in the visible region. Nano Lett..

[72] Dou K., Xie X., Pu M., Li X., Ma X., Wang C., Luo X. (2020). Off-axis multi-wavelength dispersion controlling metalens for multi-color imaging. Opto-Electron. Adv..

[73] Li K., Guo Y., Pu M., Li X., Ma X., Zhao Z., Luo X. (2017). Dispersion controlling meta-lens at visible frequency. Opt. Express.

[74] Li Y., Li X., Pu M., Zhao Z., Ma X., Wang Y., Luo X. (2016). Achromatic flat optical components via compensation between structure and material dispersions. Sci. Rep..

[75] Phan T., Sell D., Wang E.W., Doshay S., Edee K., Yang J., Fan J.A. (2019). High-efficiency, large-area, topology-optimized metasurfaces. Light Sci. Appl..

[76] Chung H., Miller O.D. (2020). High-NA achromatic metalenses by inverse design. Opt. Express.

[77] Chen W.T., Zhu A.Y., Capasso F. (2020). Flat optics with dispersion-engineered metasurfaces. Nat. Rev. Mater..

[78] She A., Zhang S., Shian S., Clarke D.R., Capasso F. (2018). Adaptive metalenses with simultaneous electrical control of focal length, astigmatism, and shift. Sci. Adv..

[79] Kamali S.M., Arbabi E., Arbabi A., Horie Y., Faraon A. (2016). Highly tunable elastic dielectric metasurface lenses. Laser Photonics Rev..

[80] Ee H.-S., Agarwal R. (2016). Tunable metasurface and flat optical zoom lens on a stretchable substrate. Nano Lett..

[81] Arbabi E., Arbabi A., Kamali S.M., Horie Y., Faraji-Dana M., Faraon A. (2018). MEMS-tunable dielectric metasurface lens. Nat. Commun..

[82] Yin X., Steinle T., Huang L., Taubner T., Wuttig M., Zentgraf T., Giessen H. (2017). Beam switching and bifocal zoom lensing using active plasmonic metasurfaces. Light Sci. Appl..

[83] Shalaginov M.Y., An S., Zhang Y., Yang F., Su P., Liberman V., Chou J.B., Roberts C.M., Kang M., Rios C. (2021). Reconfigurable all-dielectric metalens with diffraction-limited performance. Nat. Commun..

[84] Wang Q., Rogers E.T.F., Gholipour B., Wang C.-M., Yuan G., Teng J., Zheludev N.I. (2016). Optically reconfigurable metasurfaces and photonic devices based on phase change materials. Nat. Photonics.

[85] Dong K., Hong S., Deng Y., Ma H., Li J., Wang X., Yeo J., Wang L., Lou S., Tom K.B. (2018). A Lithography-Free and Field-Programmable Photonic Metacanvas. Adv. Mater..

[86] Hu T., Tseng C.-K., Fu Y.H., Xu Z., Dong Y., Wang S., Lai K.H., Bliznetsov V., Zhu S., Lin Q. (2018). Demonstration of color display metasurfaces via immersion lithography on a 12-inch silicon wafer. Opt. Express.

[87] Ahn S.H., Guo L.J. (2009). Large-area roll-to-roll and roll-to-plate nanoimprint lithography: A step toward high-throughput application of continuous nanoimprinting. ACS Nano.

[88] Yoon G., Kim K., Kim S.-U., Han S., Lee H., Rho J. (2021). Printable nanocomposite metalens for high-contrast near-infrared imaging. ACS Nano.

[89] Yoon G., Kim K., Huh D., Lee H., Rho J. (2020). Single-step manufacturing of hierarchical dielectric metalens in the visible. Nat. Commun..

[90] Yang Y., Yoon G., Park S., Namgung S.D., Badloe T., Nam K.T., Rho J. (2021). Revealing Structural Disorder in Hydrogenated Amorphous Silicon for a Low-Loss Photonic Platform at Visible Frequencies. Adv. Mater..

[91] Meem M., Majumder A., Banerji S., Rodriguez B.S., Menon R. (2021). Achromatic Broadband Visible Imaging with a 10cm Flat Lens. Proceedings of the CLEO: Applications and Technology.

[92] Lee Y.U., Li S., Bopp S.E., Zhao J., Nie Z., Posner C., Yang S., Zhang X., Zhang J., Liu Z. (2021). Unprecedented Fluorophore Photostability Enabled by Low-Loss Organic Hyperbolic Materials. Adv. Mater..

[93] Jang M., Horie Y., Shibukawa A., Brake J., Liu Y., Kamali S.M., Arbabi A., Ruan H., Faraon A., Yang C. (2018). Wavefront shaping with disorder-engineered metasurfaces. Nat. Photonics.

[94] Pahlevaninezhad H., Khorasaninejad M., Huang Y.-W., Shi Z., Hariri L.P., Adams D.C., Ding V., Zhu A., Qiu C.-W., Capasso F. (2018). Nano-optic endoscope for high-resolution optical coherence tomography in vivo. Nat. Photonics.

[95] Arbabi E., Li J., Hutchins R.J., Kamali S.M., Arbabi A., Horie Y., Van Dorpe P., Gradinaru V., Wagenaar D.A., Faraon A. (2018). Two-photon microscopy with a double-wavelength metasurface objective lens. Nano Lett..

[96] Li Z., Lin P., Huang Y.-W., Park J.-S., Chen W.T., Shi Z., Qiu C.-W., Cheng J.-X., Capasso F. (2021). Meta-optics achieves RGB-achromatic focusing for virtual reality. Sci. Adv..

[97] Lee G.-Y., Hong J.-Y., Hwang S., Moon S., Kang H., Jeon S., Kim H., Jeong J.-H., Lee B. (2018). Metasurface eyepiece for augmented reality. Nat. Commun..

[98] Rubin N.A., D’Aversa G., Chevalier P., Shi Z., Chen W.T., Capasso F. (2019). Matrix Fourier optics enables a compact full-Stokes polarization camera. Science.

[99] Guo Q., Shi Z., Huang Y.-W., Alexander E., Qiu C.-W., Capasso F., Zickler T. (2019). Compact single-shot metalens depth sensors inspired by eyes of jumping spiders. Proc. Natl. Acad. Sci. USA.

[100] Holsteen A.L., Lin D., Kauvar I., Wetzstein G., Brongersma M.L. (2019). A light-field metasurface for high-resolution single-particle tracking. Nano Lett..

[101] Wei S., Cao G., Lin H., Yuan X., Somekh M., Jia B. (2021). A Varifocal Graphene Metalens for Broadband Zoom Imaging Covering the Entire Visible Region. ACS Nano.

[102] Aiello M.D., Backer A.S., Sapon A.J., Smits J., Perreault J.D., Llull P., Acosta V.M. (2019). Achromatic varifocal metalens for the visible spectrum. ACS Photonics.

[103] Fan C.-Y., Chuang T.-J., Wu K.-H., Su G.-D.J. (2020). Electrically modulated varifocal metalens combined with twisted nematic liquid crystals. Opt. Express.

[104] Qin S., Xu N., Huang H., Jie K., Liu H., Guo J., Meng H., Wang F., Yang X., Wei Z. (2021). Near-infrared thermally modulated varifocal metalens based on the phase change material Sb2S3. Opt. Express.

[105] Joo W.-J., Kyoung J., Esfandyarpour M., Lee S.-H., Koo H., Song S., Kwon Y.-N., Song S.H., Bae J.C., Jo A. (2020). Metasurface-driven OLED displays beyond 10,000 pixels per inch. Science.

[106] Kim I., Kim W.-S., Kim K., Ansari M.A., Mehmood M.Q., Badloe T., Kim Y., Gwak J., Lee H., Kim Y.-K. (2021). Holographic metasurface gas sensors for instantaneous visual alarms. Sci. Adv..

